# Nuclear Multi‐Microsatellite Marker Profiling Provides Clues to Molecular Genetic Diversity in Culture‐Based Caspian Beluga Sturgeon (*Huso huso*) Brood Stocks: Ecological Mirror for Restoration

**DOI:** 10.1002/vms3.70255

**Published:** 2025-04-08

**Authors:** Mehdi Moghim, Arash Javanmard, Faramarz Lolaei, Mohammad‐Javad Taghavi, Shima Bakhshalizadeh

**Affiliations:** ^1^ Department of Genetics Caspian Sea Ecology Research Centre Sari Iran; ^2^ Department of Animal Science Faculty of Agriculture University of Tabriz Tabriz Iran; ^3^ Department of Stock Assessment Caspian Sea Ecology Research Centre Sari Iran; ^4^ Department of Marine Science Caspian Sea Basin Research Center University of Guilan Rasht Iran

**Keywords:** beluga sturgeon, broodstocks, Caspian sea, genetic diversity, microsatellite

## Abstract

The decreasing natural genetic diversity within intense beluga sturgeon aquaculture presents a complex challenge for culture‐based sturgeon stocks. The present report aims to assess the situation by utilizing advanced capillary electrophoresis (CE) and multi‐microsatellite nuclear marker tools. We conducted a study involving 436 individuals of both sexes, collected from eight breeder private farms with diverse breeding histories and generational backgrounds. Through the application of eight microsatellites, we amplified a non‐coding core genomic region in the species, followed by CE to provide double confirmation of observed genotype and actual allelic size SSR profiling. Utilizing molecular descriptive statistics in the POPGENE software, we calculated allele frequencies, expected and observed heterozygosity within populations, the number of observed and effective alleles (*n_a_* and *n_e_*) and the Shannon's Information Index. Furthermore, we performed molecular analysis of variance (AMOVA), model‐based clustering, principal coordinate analysis (PCoA) and STRUCTURE analysis to genetically characterize the populations. It was revealed that LS19 (*n_a_* = 16) and Afu54 (*n_a_* = 3) exhibited the highest and lowest levels of polymorphisms, respectively, within the studied families. Moreover, the Iranian Fisheries Research Organization (IFRO) farm population was found to have the highest genetic diversity (Ave_Het = 0.67), whereas the Rajaeei Sturgeon Private Farm (RJI) displayed the lowest diversity score (Ave_Het = 0.55) among the examined populations. INS and Jahanpouri Sturgeon Private Farm (JPR) showed the highest similarity (0.91), whereas the Saeei Sturgeon Private Farm had the lowest genetic distance, with a similarity score of 0.74 among the populations studied. Furthermore, the evidence from the STRUCTURE analysis highlighted notable levels of allelic sharing and admixture among the eight studied populations, indirectly indicating the presence of genetic diversity within each population and the relatively low genetic distance between the populations. The results demonstrate a significant level of genetic variability, providing evidence that supports the low value of inbreeding in brood management.

## Graphical abstract

We conducted a study involving 436 individuals of both sexes, collected from eight breeder private farms with diverse breeding histories and generational backgrounds. Through the application of eight microsatellites, we amplified a non‐coding core genomic region in the species, followed by capillary electrophoresis (CE) to provide double confirmation of observed genotype and actual allelic size SSR profiling. Utilizing molecular descriptive statistics in the POPGENE software, we calculated allele frequencies, expected and observed heterozygosity within populations, the number of observed and effective alleles (*n_a_* and *n_e_*) and the Shannon's Information Index. Furthermore, we performed molecular analysis of variance (AMOVA), model‐based clustering, principal coordinate analysis (PCoA) and STRUCTURE analysis to genetically characterize the populations.

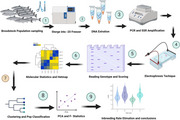

**Abbreviations**
F: F‐statistics
*H_e_*
: expected heterozygosity
*H_o_*
: observed heterozygosity
I: Shannon index
*N*
: number of samples
*N_a_*
: observed allele
*N_e_*
: expected allele
*uH_e_*
: mean of heterozygosity

## 1 Introduction

The beluga sturgeon (*Huso huso*), also known as the giant sturgeon, is a globally valued freshwater species belonging to the distinguished family Acipenseridae (Kohlmann et al. 2018). The beluga sturgeon can grow up to 6 m in length and weigh over 2000 kg, making it one of the largest freshwater fish species. Although the sexes of this species share similar morphological characteristics, females typically exhibit larger physical dimensions (Ludwig et al. 2008; Williot et al. 2011). Moreover, the beluga sturgeon holds significant economic importance as a primary source of caviar, meat and resources for aquaculture, representing an endemic species within the Caspian Basin (Bakhshalizadeh et al. 2021).

Like other sturgeon species, beluga sturgeon is characterized by traits that enable adaptation to various environmental conditions delayed maturation, longevity and high fertility, traits that enable adaptation to various environmental conditions. Sturgeons typically undergo a protracted life cycle, reaching sexual maturity at advanced ages, usually between 10 and 18 years, and spawning at intervals of 3–5 years (Onara et al. 2014). The roe of the beluga sturgeon, known as caviar, is highly valued in international markets, with prices exceeding $5000 per kilogram in the United States. Furthermore, the meat production of this fish garners significant interest in multiple countries (Norouzi and Pourkazemi 2016).

The great sturgeon population has sharply declined due to river poaching, leading to little natural reproduction. This has resulted in recruitment mainly relying on breeding stock (Boscari et al. 2021). Sustainable sturgeon management in Iran faces challenges from human activities such as habitat loss, overfishing, poaching, illegal trade and pollution (Moghim et al. 2009; Khoshkholgh et al. 2011; Moghim et al. 2012; Beridze et al. 2022). In Romania, studies have highlighted genetic diversity threats in the Black Sea beluga sturgeon populations due to overexploitation and habitat changes (Dudu et al. 2008). Similarly, in Russia, significant efforts are ongoing to restore the beluga sturgeon populations in the Azov Sea basin, reflecting a regional response to overfishing and habitat degradation (Rudoy et al. 2024). Furthermore, in Turkey, the recent discovery and subsequent death of a beluga sturgeon in the Küçükçekmece Lagoon underscore the importance of addressing pollution and habitat degradation in the region (Memiş et al. 2024). Incorporating these studies (Dudu et al. 2008; Rudoy et al. 2024; Memiş et al. 2024) into this section would not only strengthen the manuscript's global perspective but also illustrate that the threats to sustainable sturgeon management are a shared issue requiring coordinated international efforts. This broader context will make the manuscript more relevant to a wider audience and enhance its scientific impact.

Additionally, high levels of heavy metals and organic compounds in the Caspian Sea can hinder reproduction (Ludwig et al. 2000). Sturgeon hatcheries are vital for replenishing sturgeon populations (Zhang et al. 2013). However, limitations in restoration efforts stem from the lack of information about the genetic diversity of released cohorts and the inability to determine if a wild animal originated from a hatchery.

The recovery of wild populations heavily relies on producing highly genetically selected offspring and releasing them into the Caspian Sea (Norouzi and Pourkazemi 2016). Therefore, understanding the genetic makeup of culture‐based stocks is essential for developing strategies to safeguard the beluga sturgeon in the Caspian Sea (Moghim et al. 2009). The genetic diversity of the population includes both inter‐ and intra‐variation, and this diversity enables certain species to adapt flexibly to diverse habitats. In the genetic management of marine animals, genetic markers have been utilized for many years to analyse fish stock dynamics and inform management decisions (Fopp‐Bayat 2010). Genetic markers can help determine genetic variation among individuals within populations or between aquaculture and natural populations (Zhao et al. 2005; Antognazza et al. 2021).

Both theoretical and empirical studies indicate that genetic diversity is diminished in small populations due to the combination of random genetic drift and breeding among closely related individuals (Allendorf et al. 2013). The decline in genetic variation and the subsequent increase in homozygosity can lead to the expression of detrimental recessive alleles and a decrease in fitness. Microsatellite markers are polymorphic loci found in both nuclear and organelle DNA, containing repeating units 14 base pairs in length. These markers are typically neutral and co‐dominant and are widely used in population genetics for various applications, including kinship and population studies (Dudu et al. 2014). Microsatellite markers, and more recently, the use of single nucleotide polymorphisms (SNPs), have become preferred for genetic inventory delineation, species phylogeography, hybridization identification, translocation pathway determination and the identification of genetic traits of interest, among other applications (Wang et al. 2022). Numerous studies have been conducted to address genetic diversity issues in sturgeon populations (Forlani et al. 2008; Börk et al. 2008; Dudu et al. 2011; Chassaing et al. 2011).

Maintaining genetic diversity in breeding population production and subsequent generations of captive breeding and selection is crucial to prevent inbreeding and the resulting decline in fitness and performance traits (Brown et al. 2009). In cases where the breeding stock population exhibits moderately high genetic diversity, crosses between different strains can serve as a valuable starting point for establishing a highly variable base population for selective breeding activities (Allendorf et al. 2013). To safeguard the genetic health of sturgeon spawn, breeding managers can use an appropriate number of founders for producing subsequent generations and trace pedigrees using physical or genetic markers to prevent mating among close relatives (Welsh et al. 2003).

It is crucial to comprehend the complexities and factors influencing the survival and conservation of the beluga population structure. As such, an understanding of DNA variability and population structure is fundamental and forms an essential component of any conservation programme, providing insights into the species’ biology. In this report, our focus has been on monitoring and measuring genetic diversity in breeding populations of Caspian Beluga sturgeon (*H. huso*) using microsatellite markers and advanced capillary electrophoresis genotyping tools.

## 2 Materials and Methods

### 2.1 Fish and Studied Farms

**FIGURE 1:**
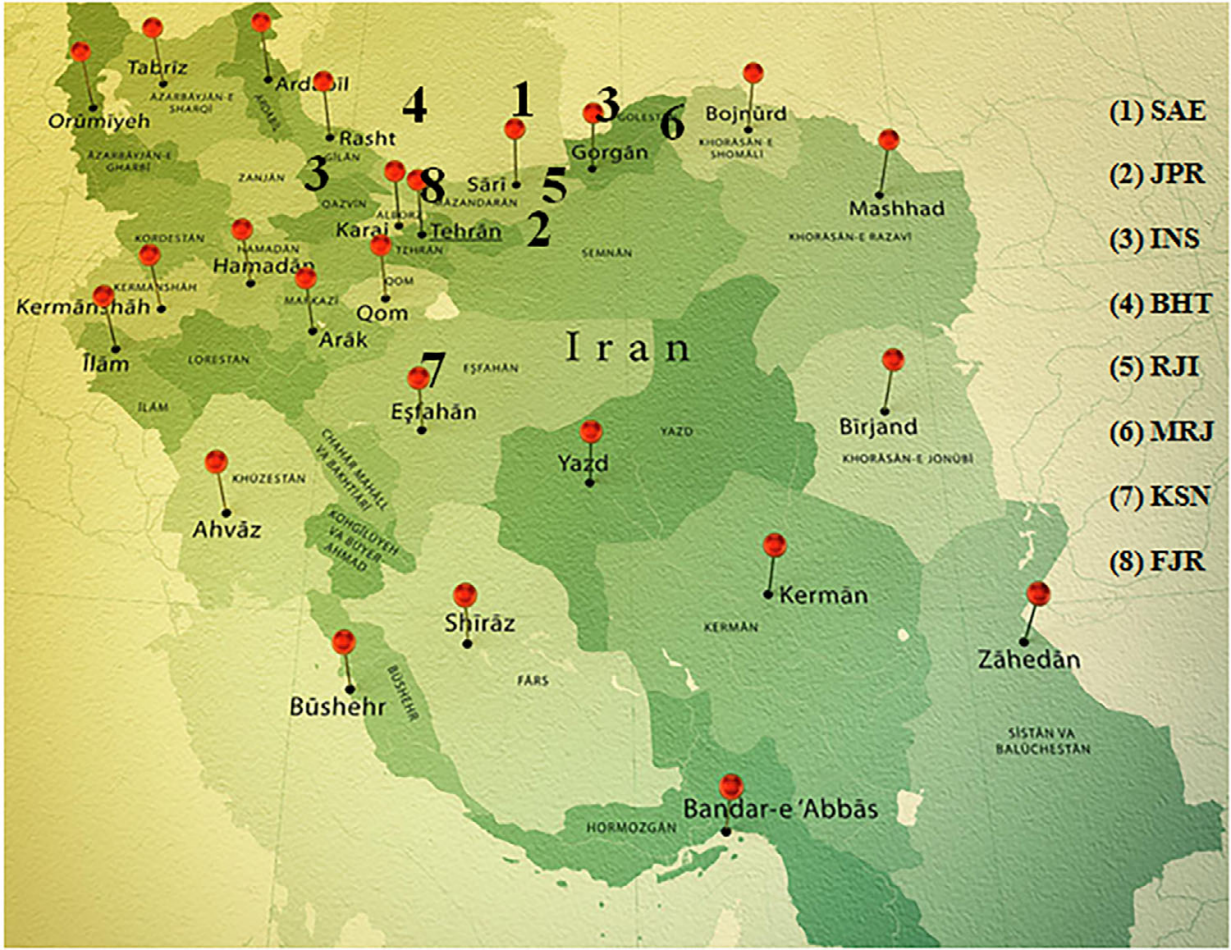
Iranian Fisheries Research Organizations and private fish farms.

**FIGURE 2:**
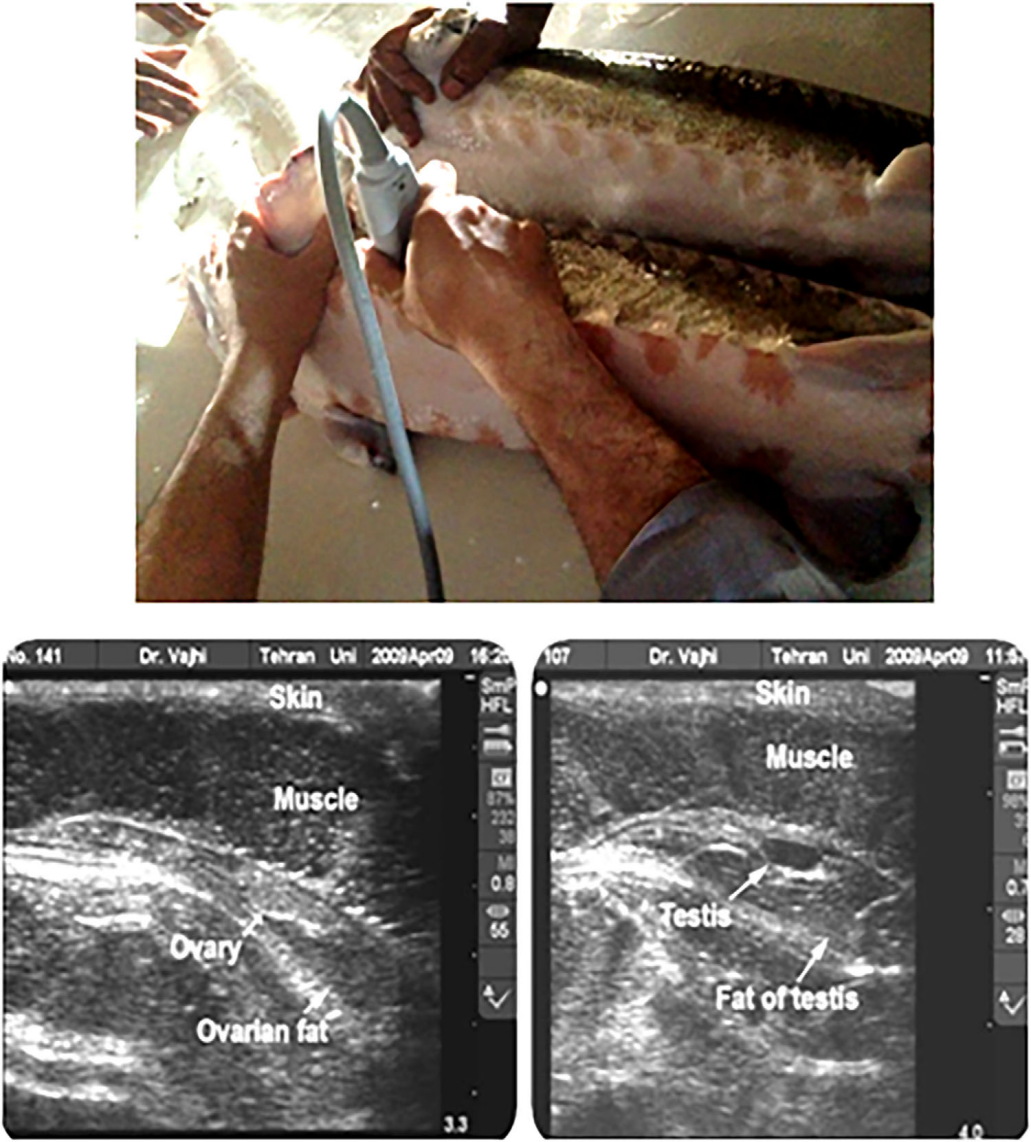
Determination of the gender in each broodstock sturgeon farm based on ultrasound method.

For this study, we randomly gathered 424 beluga sturgeon of both sexes from 8 Iranian Fisheries Research Organizations (IFROs) and private fish farms (Figure 1). These broodstocks were housed together for spawning in flow aquaculture systems at the company's hatchery. The determination of gender in each broodstock sturgeon farm is based on a non‐invasive ultrasound method as described by Moghim et al. (2002) (Figure 2). Table 1 provides detailed information on the eight beluga sturgeon breeding populations studied. Behashti Sturgeon Private Farm (BHT) (average weight 8–10 kg), Marjani Sturgeon Private Farm (MRJ) (average weight 13–20 kg), Rajaeei Sturgeon Private Farm (RJI) and IFRO are the four main distributors of broodstock to other farmers and geographical areas for collecting individuals. Some of the broodstock populations experienced F1, and some had F2–F3 generations during our sampling period.

**TABLE 1:** Overview of the studied population.

Sturgeon private farm	ID	Province	Female	Male
Marjani	MRJ	Gholestan	15	15
Rajaeei	RJI	Mazandaran	40	12
Behashti	BHT	Giluan	30	22
Iranian Fisheries Research Organization	INS	Giluan	32	15
Saeei	SAE	Mazandaran	31	30
Jahanpouri	JPR	Mazandaran	30	32
Kashan	KSN	Isfahan	30	30
Fajr Amol	FJR	Mazandaran	30	30
				424

Note: Studied populations are experiencing variable generation numbers during the sampling process and some breeder farmers had exchange plans between their broodstocks.

The fish larvae from the RJI private farm originated from broodstock propagated from the sea fry of the private BHT in Rasht, and subsequently transferred to the private RJI farm. The JPR farm fish larvae were derived from sea‐propagated broodstock fish from the private MRJ Hatchery in Gorgan City and transported to Babolsar city for rearing. The fish larvae at the private BHT Farm were artificially bred and raised for the production of the private RJI. In the workshop of private RJI, 150–200 broodstocks from the farm's artificial propagation for the production of Sari City are kept and raised annually. At the private MRJ Farm, the selected breeders, aged 9–11 years, have been maintaining and raising artificially propagated broodstock for the private RJI Breeder Centre for several years. The fish larvae at the private KSN were transferred and raised from a farm in Qom, whereas the broodstock at the private FJR Farm was artificially bred, brought and raised from the city of Gorgan and part of the Qom area.

### 2.2 Finclips Collection, DNA Extraction and Microsatellite Genotyping

After the required samples were selected and cut, the caudal hair root was stored at 4°C. Individual hairs were randomly removed with tweezers, and the presence of a root mark was visually checked.

In this study, we carried out the extraction of total genomic DNA from fin clips, which were approximately 0.5 cm^3^ in size, using the Qiagen DNeasy kit (Qiagen, Valencia, CA). The Touchdown PCR program and a routine electrophoresis protocol were implemented as outlined in the molecular cloning textbook manual (May et al. 1997; McQuown et al. 2003). We employed eight microsatellites to amplify a non‐coding core genomic region in this species. The primer sequences and annealing temperatures for these marker loci can be found in Table 2.

**TABLE 2:** Primer sequences and annealing temperatures for these three marker loci.

Loci	Motif	Primer sequence	Accession number	Expected size (bp)
**Ape‐20**	(GACA)5	CACTGCCTGCTGCCTAAAAC ACTGTGGGGCTCTGTCTGTC	EU531732	176
**LS‐19**	(TTG)9	CATCTTAGCCGTCTGTGGTAC CAGGTCCCTAATACAATGGC	U72730	125–164
**LS‐39**	(GTT)10	TTCTGAAGTTCACACATTG ATGGAGCATTATTGGAAGG	U72734	129
**LS‐54**	(GATA)6	CTCTAGTCTTTGTTGATTACAG CAAAGGACTTGAAACTAGG	U72735	177
**Afu‐68**	(GATA)13	TTATTGCATGGTGTAGCTAAAC AGCCCAACACAGACAATATC	OV754680.1	120–140
**Afu‐54**	(GATA) 6(GACA) 7	CTCTAGTCTTTGTTGATTACAG CAAAGGACTTGAAACTAGG	U72735.1	160–180
**Spl‐120**	(TATC) 15	ATTCCATGAGCAACACCACA TGATGGTCTGATGAGATCGG	AF276189	263–303
**Afu‐G9**	((GATA)14(GA)2GATA (GA)2(GATA)6	CATAATGTAAAGCAAAAGT ACCTGAAATGTATGTTATG	AF529447	130–160

*Note*: All of our studied loci showed genotype within range of pervious literatures.

The microsatellite alleles were scored manually by comparing them with allelic ladders run on each gel. The PCR reaction consisted of a total volume of 25 µL, including 0.1 mM dNTP, 2.5 mM MgCl_2_, 0.5 mM of each specific primer, PCR buffer (1X), 2 U Taq polymerase (Denmark) and 50 ng DNA template. The PCR machine model used for amplifying the fragment was the Biometra T‐Gradient. The PCR programme comprised an initial denaturation phase at 94°C for 8 min, followed by 10 cycles with a decreasing gradient starting at a temperature of 68°C and ending at 52°C. The specific details for each cycle included an initial step at 95°C for 1 min, followed by a temperature drop specific to each cycle for 1 min and a reproduction temperature at 72°C for 1 min. The last cycle involved denaturation at 95°C for 1 min, a reduction temperature of 58°C for 1 min and a reproduction temperature of 72°C for 1 min. Finally, the final reproduction temperature was set to 72°C for 8 min.

The pre‐assay of the pooled loci involved horizontal electrophoresis on 8% Metaphor agarose gels for 40 min in 2.5% low melting point agarose gels, which were stained with RedSafe dye (iNtRON Inc.) and visualized under UV light. The power supply model utilized for electrophoresis was PAC1000 from Bio‐Rad company, USA. Additionally, the estimation of PCR product size by gel was conducted using a standard size marker ladder of 100 bp and 1 kb (Fermentas, USA) with the assistance of the BIO 1D^++^ computer software.

### 2.3 Fragment Analysis Based on Capillary Electrophoresis (CE)

CE is a powerful method for SSR profiling due to its ability to provide highly accurate allele sizes and genotypes while minimizing stuttering‐unspecific bands. After evaluating the PCR products using conventional gel electrophoresis, we transferred the amplicons to CE for further analysis. To prepare the samples, we used a 20 µL multichannel pipette to transfer the PCR products (0.4–1.0 µL each) to the CEQ sample plate. We then labelled the forward primers with WellRED dye (Beckman Coulter Inc.), mixed 0.9 µL PCR product with 30 µL sample loading solution (SLS) and added 0.25 µL 400 bp size standard (D1, red) along with 0.1 mL of Sigma mineral oil. The prepared samples were then applied to the plate and briefly centrifuged.

After the electrophoretic separation of all samples in one plate was completed, we rinsed out the contents of the capillary, filled the capillaries with fresh matrix and loaded them into a Beckman Coulter Inc. genetic analysis system (plate_number_1). We followed the instructions displayed on the screen for further analysis.

### 2.4 Statistical Analysis

Molecular descriptive statistics in the POPGENE software were used to calculate allele frequencies, expected and observed heterozygosity within populations, the number of observed and effective alleles (*n_a_* and *n_e_*) and the Shannon's Information Index (Yeh 1999). Additionally, molecular analysis of variance (AMOVA), model‐based clustering, principal coordinate analysis (PCoA) and STRUCTURE analysis were performed to genetically characterize the populations (Pritchard et al. 2000; Excoffier et al. 2005). To determine the optimal number of clusters (*K*), the population structure was tested at *K* values ranging from 1 to 8 with 10 iterations. Each iteration was based on 100,000 iterations of the Markov Chain Monte Carlo (MCMC) after a burn‐in period of 10,000 steps. Subsequently, the optimal *K* was evaluated using STRUCTURE HARVESTER v6.94.

## 3 Results

### 3.1 Microsatellite Genotyping Based on CE

**FIGURE 3:**
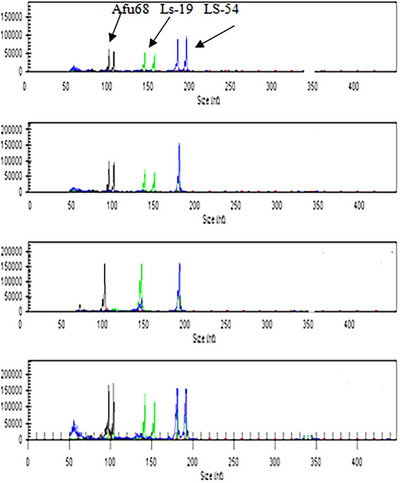
Overview of capillary electrophoresis graph for triplex loci within studied populations.

The results depicted in Figure 3 present the outcomes of CE for triplex loci in four individuals. The application of CE is demonstrated to effectively mitigate genotyping errors and provide precise estimates of allelic sizes. Furthermore, this technique can accurately discriminate alleles with variances of 2–4 base pairs, thereby establishing a more dependable and accurate method for discerning the number of alleles compared to traditional routine methods.

### 3.2 Molecular Descriptive Statistics for Used Candidate Primers

**FIGURE 4:**
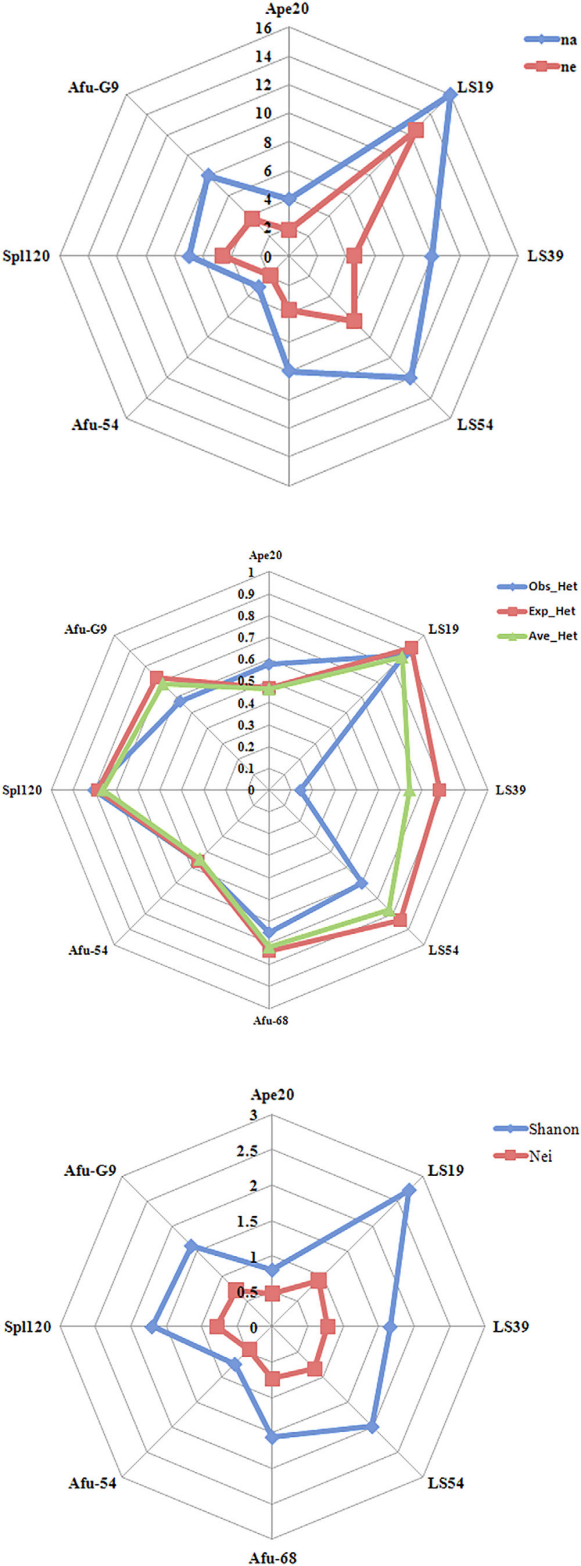
Snapshot of molecular diversity parameters for each primer across the population. *n_a_*: observed allele; *n_e_*: effective allele; Mean (Ne)_: Mean Nei Index; Mean (Het): Mean of observed heterozygosity.

Furthermore, the population from the IFRO farm exhibited the highest genetic diversity, as evidenced by average heterozygosity (Ave_Het) of 0.67, whereas the Rajaeei Sturgeon Private Farm (RJI) population displayed the lowest genetic diversity, with an average heterozygosity of 0.55. Figure 4 offers a comprehensive portrayal of the molecular diversity parameters for each locus, providing a nuanced perspective on polymorphism across the studied populations. Additionally, the presence of heterozygosity in the population serves as an indicator of sustained long‐term natural selection, marking the organism's adaptation to natural conditions.

### 3.3 Genetic Diversity Within Studied Populations

**FIGURE 5:**
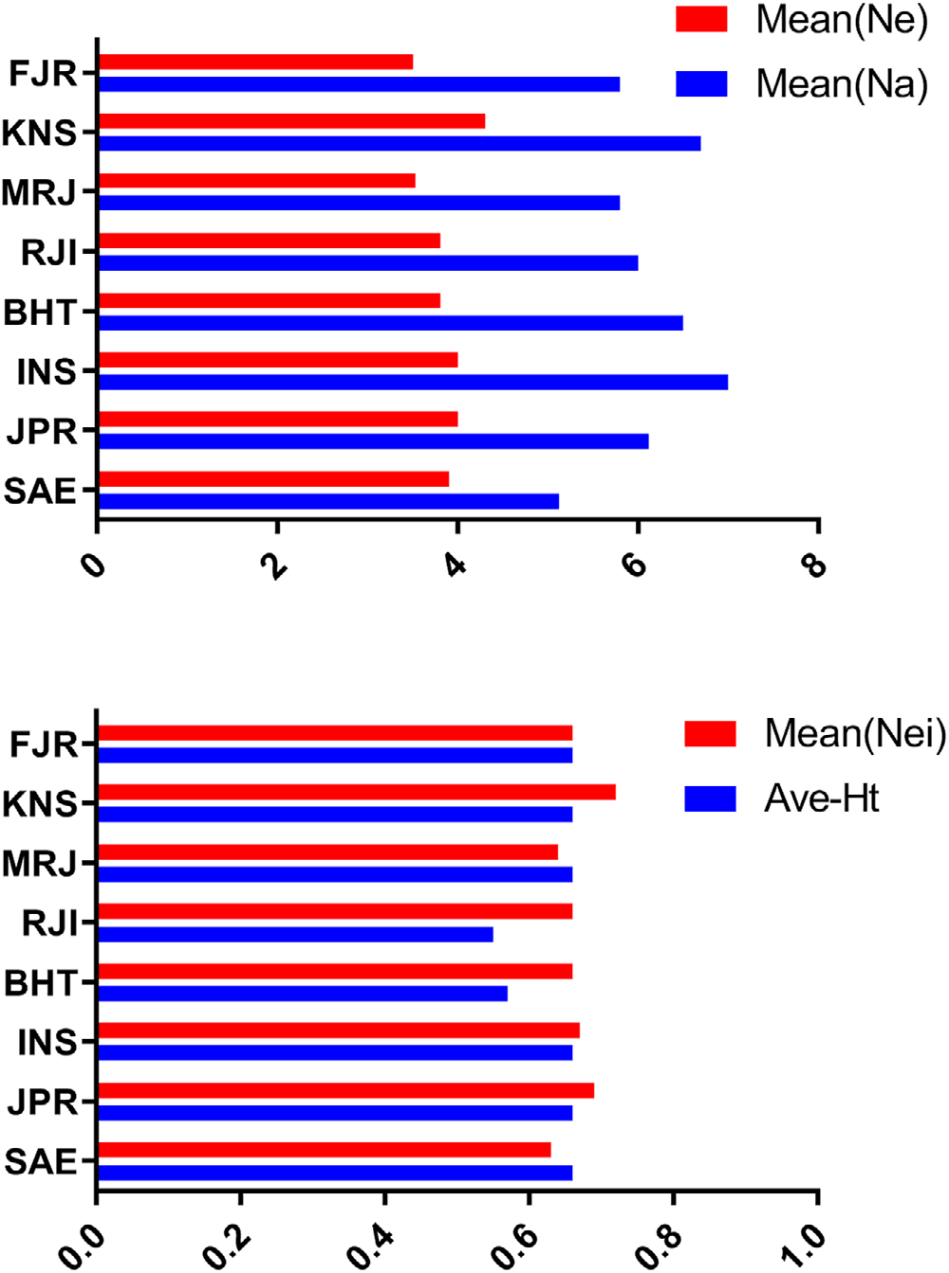
Summary of statistics for genetic diversity within studied populations. *n_a_*: observed allele; *n_e_*: effective allele Studied Sturgeon Private Farm: Marjani (MRJ); Rajaeei (RJI); Behashti (BHT); Iranian Fisheries Research Organization (INS); Saeei (SAE); Jahanpouri (JPR); Kashan (KSN); Fajr Amol (FJR).

The analysis of the mean molecular parameters (*n_a_*, Obs_het, Nei and Ave_het) for the eight studied populations unveiled a consistent pattern of diversity. Our study further revealed that the INS and Jahanpouri Sturgeon Private Farm (JPR) exhibited the highest similarity (0.91), indicating a close genetic relationship, whereas the Saeei Sturgeon Private Farm showed the lowest genetic distance (similarity 0.74) among the studied populations. Figure 5 provides a comprehensive statistical summary detailing the genetic diversity within the studied populations, offering valuable insights into the similarities and differences observed.

### 3.4 Genetic Diversity Between Studied Populations

**FIGURE 6:**
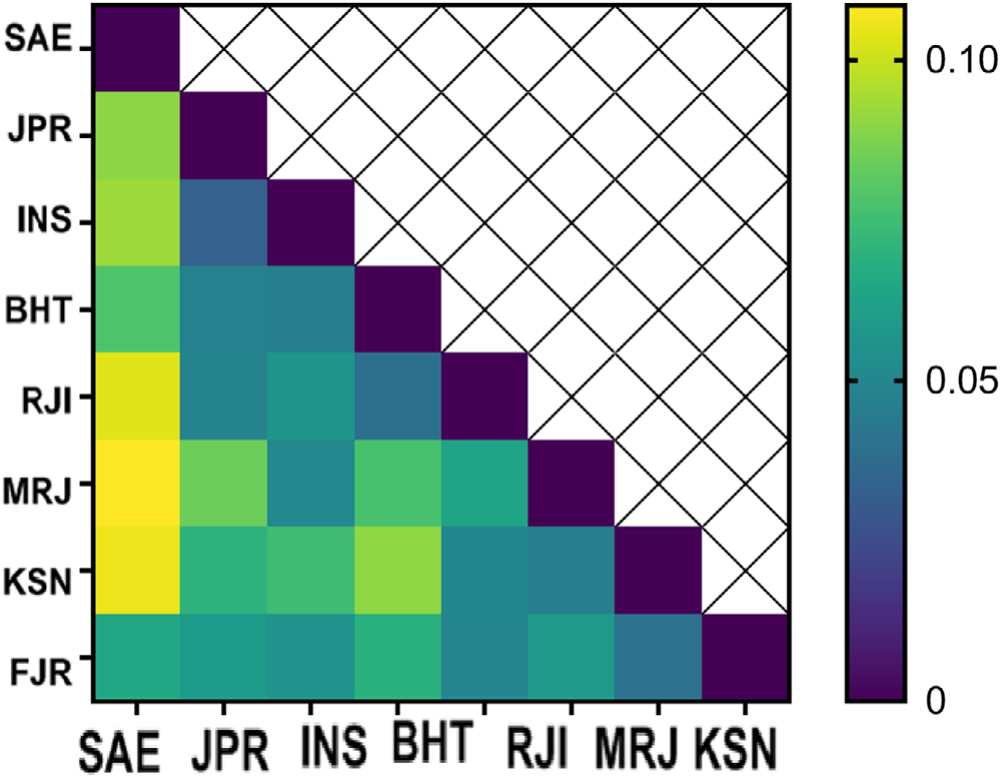
Overview of genetic similarity and distance among eight studied populations. Studied Sturgeon Private Farm: Marjani (MRJ); Rajaeei (RJI); Behashti (BHT); Iranian Fisheries Research Organization (INS); Saeei (SAE); Jahanpouri (JPR); Kashan (KSN); Fajr Amol (FJR).

**FIGURE 7:**
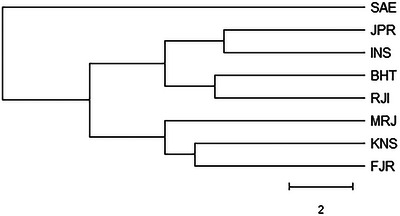
Cluster‐based evidence for genetic distance value for eight studied populations. Studied Sturgeon Private Farm: Marjani (MRJ); Rajaeei (RJI); Behashti (BHT); Iranian Fisheries Research Organization (INS); Saeei (SAE); Jahanpouri (JPR); Kashan (KSN); Fajr Amol (FJR).

**FIGURE 8:**
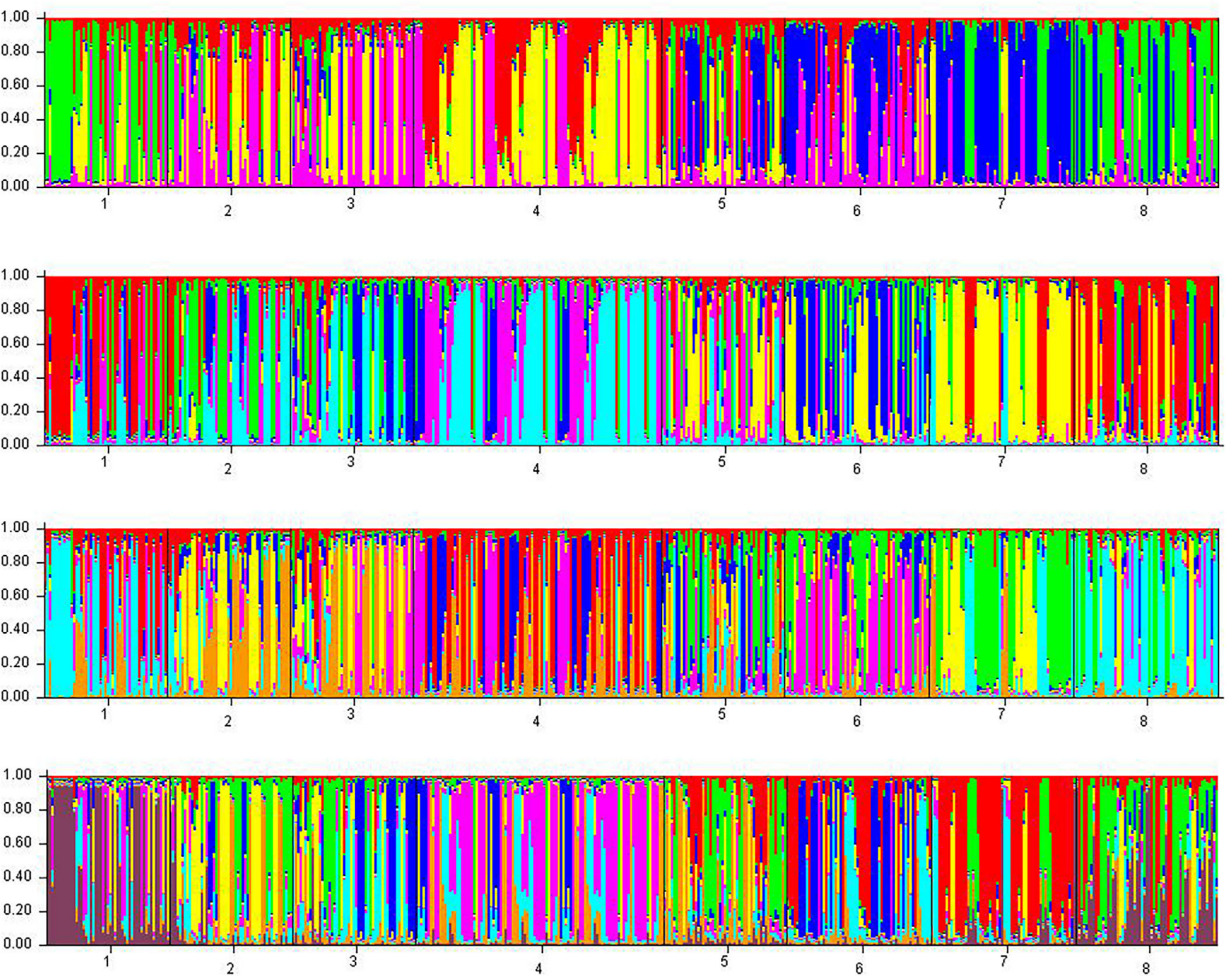
Structure and Bayesian clustering based on allele sharing and allele admixture for eight studied populations. Studied Sturgeon Private Farm: Marjani (MRJ); Rajaeei (RJI); Behashti (BHT); Iranian Fisheries Research Organization (INS); Saeei (SAE); Jahanpouri (JPR); Kashan (KSN); Fajr Amol (FJR).

**FIGURE 9:**
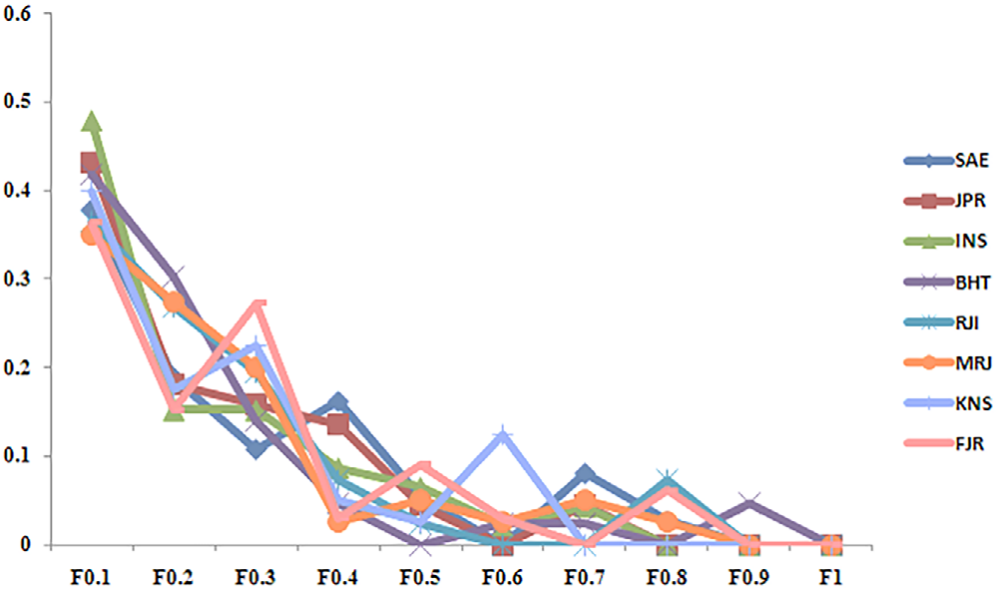
Summary of Bottleneck‐based analysis for eight studied populations.

Our research findings revealed that the INS and MRJ farms exhibited the highest similarity (0.97), whereas the Saeei Sturgeon Private (SAE) farms showed the highest genetic distance (similarity 0.89) among the populations under study. Additionally, the comparison between SAE and KSN indicated the highest genetic distance value (0.70 similarities) among the studied populations. Figure 6 provides an overview of the genetic similarity and distance across the eight studied populations, whereas Figure 7 presents cluster‐based evidence of genetic distance values. Furthermore, Figure 8 illustrates the structure and Bayesian clustering based on allele sharing and mixing for the eight populations, and Figure 9 depicts the bottleneck analysis, indicating an l‐shaped pattern reflecting a low inbreeding rate across all studied broodstock populations.

## 4 Discussion

Understanding the biology and reproduction of sturgeon, including their spawning and migration behaviours, is crucial for selecting and cultivating the next generation of sturgeon breeders in artificial ponds. This selection process aims to maintain diversity and introduce new alleles through diverse mating pairs, thus ensuring genetic diversity (Dudu et al. 2014). The genetic diversity and population genetic structure in sturgeon are influenced by various factors such as geographical location, environmental conditions, selective forces, primary stock status, mating modes and the interaction between captive and wild populations. It is essential to acknowledge that a reduction in genetic diversity can hinder populations’ ability to adapt to environmental pressures such as climate change or changes in available resources (Wang et al. 2022; Dudu et al. 2014). Reduced genetic diversity implies the potential loss of underlying genetic diversity crucial for adaptation. Heterozygotes, carrying different alleles, contribute to diversity and adaptation to environmental conditions, influencing economic, fertility and disease resistance traits (Wang et al. 2022). The number of alleles and heterozygosity can also be influenced by the sample size and the target region.

In the realm of culture‐based sturgeon stocks, a pressing concern pertains to the diminishing natural genetic diversity within intense beluga sturgeon aquaculture. Preserving a substantial level of genetic diversity within the population is crucial to mitigate inbreeding and genetic drift. In line with this objective, we conducted an assessment of genetic diversity in culture‐based breeding populations of the *H. huso* species utilizing a multi‐microsatellite nuclear marker via CE.

### 4.1 Molecular Descriptive Statistics for Used Candidate Primers

Our analysis revealed that among the families examined, LS19 (*n_a_* = 16) and Afu54 (*n_a_* = 3) demonstrated the highest and lowest levels of polymorphisms, respectively. Furthermore, similar SSR patterns and allele sizes have been documented in previous studies by Forlani et al. (2008), Börk et al. (2008), Dudu et al. (2011), Chassaing et al. (2011), and Barmintseva and Mugue (2013). The high number of alleles is indicative of greater genetic variation (Saitou and Nei 1987) and holds significant importance for conservation programmes. Although these findings elucidate the prevalence of high polymorphism, it is crucial to note that the average number of alleles is influenced by the sample size, with the observed number of alleles typically increasing with larger population sizes (Aljumaah et al. 2012). Consequently, it is essential to select population sizes that are relatively equivalent for comparison purposes. However, it is worth noting that some studies have utilized a very small number of animals, contrary to the FAO recommendation for microsatellite marker analysis.

### 4.2 Genetic Diversity Within Studied Populations

In addition, the IFRO farm population (Ave_Het = 0.67) exhibited the highest genetic diversity, whereas the Rajaeei Sturgeon Private Farm (RJI) (Ave_Het = 0.55) demonstrated the lowest diversity score among the populations under study. Our current findings align with previous research conducted by Fopp‐Bayat (2010) on the Siberian sturgeon, *Acipenser baerii*, using 6 SSR loci, which reported 812 alleles per locus and a genetic diversity of 0.680. Similarly, Ludwig et al. (2000) examined *Acipenser sturio* using 5 SSR markers and found 14–22 alleles per locus. Zhao et al. (2005) studied the Chinese sturgeon (*Acipenser sinensis* Grey) with 4 SSR, reporting 4–15 alleles per locus and a genetic diversity score ranging from 0.34 to 0.67. Reduced levels of heterozygosity can be attributed to isolation and the consequent loss of genetic potential. The heterozygosity of a gene locus correlates with the polymorphic nature of each locus, with higher heterozygosity rates typically associated with greater genetic diversity, ultimately facilitating species adaptation to natural conditions. The degree of heterozygosity at gene loci serves as a critical indicator of the self‐homogeneity within populations, offering insights into gene diversity. When the Shannon index approaches 1, it suggests a high level of heterozygosity within our population. Furthermore, comparing the Shannon index calculated for different loci can provide valuable insights into the suitability of specific primers for capturing the genetic diversity of the breed or population under study. These variations can be attributed to a range of factors including distinct population histories, sample sizes, electrophoresis techniques, genetic backgrounds, SSR primers and geographic distributions within different sturgeon species when compared to findings from previous reports.

The beluga sturgeon (*H. huso*), a critically endangered species in the Caspian Sea, faces similar environmental and anthropogenic challenges across its range, including overfishing, habitat degradation and restricted migration routes. Genetic studies using microsatellite markers on both Russian and Iranian populations reveal consistent patterns of moderate genetic diversity, population differentiation and inbreeding risks. For instance, research on Russian beluga sturgeon from the Volga River and other Caspian regions has shown moderate to high genetic diversity but significant differentiation between populations, indicating limited gene flow (Mugue et al. 2016; Barmintseva et al. 2019). Similarly, studies on Iranian beluga sturgeon populations have reported moderate genetic diversity but signs of inbreeding and reduced gene flow, likely due to habitat fragmentation and overexploitation (Rezvani Gilkolaei et al. 2018). Both populations exhibit elevated inbreeding coefficients (FIS), particularly in isolated or small groups, highlighting the need for conservation strategies that minimize inbreeding and enhance genetic connectivity. These findings suggest that Russian and Iranian beluga sturgeon share common genetic challenges, reflecting the broader environmental pressures in the Caspian Sea. To address these issues, practical conservation measures such as restoring natural migration routes, implementing controlled breeding programmes and conducting long‐term genetic monitoring are essential. Additionally, transboundary cooperation between Caspian Sea countries is crucial for developing unified conservation policies and ensuring the long‐term survival of this iconic species (Pourkazemi et al. 2017).

### 4.3 Genetic Diversity Between Studied Populations

The study of population genetics involves various factors that influence the evolution and effective size of populations, such as the rate of drift, mutational selection and migration. Our findings also indicated low levels of inbreeding across all studied populations, indirectly suggesting a preference for outbreeding within the broodstock individuals. In contrast, Zhang et al. (2013) reported a genetic variance of 81.73% within populations and a strain variance of 9.40% between populations, presenting differing results. Furthermore, Norouzi and Pourkazemi (2016) provided detailed insights into genetic diversity within the studied population of star sturgeon (*Acipenser stellatus*), documenting a mean observed heterozygosity score of 0.665, a mean heterozygosity of 0.862 and a mean FIS of 0.230.

Furthermore, our study revealed that the populations INS and JPR exhibited the highest similarity with a score of 0.91, whereas the Saeei population showed the lowest genetic distance with a similarity of 0.74. This observation suggests that the diversity within the population is higher than the diversity between the populations, indicating the absence of inbreeding events within the studied population stocks. With advancements in molecular genetic techniques and the development of new devices, it has become easier to gather information about allele frequencies and the number of differences (mutations) between alleles. When examining molecular variation, it is essential to utilize haplotypic data to ensure that there are no variations within individuals (Wang et al. 2022; Dudu et al. 2014).

Our investigation revealed that the INS and MRJ farms exhibited the highest similarity, whereas the SAE farms showed the highest genetic distance among the studied populations. The cluster analysis of genetic parameters further corroborated the insights gained into the genetic diversity across the populations under study. Genetic distance, which represents the degree of genetic difference (genomic difference) between species or populations, is evaluated using a numerical method. The average number of codon or nucleotide differences per gene serves as a measure of genetic distance (Saitou and Nei 1987). The genetic distance between two populations is theoretically defined using the population gene frequencies for all loci in the genome. In practice, however, it is impractical to examine all genes in the populations for all loci. Therefore, estimating genetic distance entails sampling a certain number of individuals from the populations and examining a specific number of loci (Wang et al. 2022; Dudu et al. 2014).

Population differentiation is a key parameter in conservation genetics, as it provides insights into the genetic structure and connectivity among populations. Microsatellite markers are highly effective for measuring population differentiation due to their high polymorphism and neutrality (Selkoe and Toonen 2006). In our study, we used FST and GST statistics to quantify genetic differentiation among sturgeon populations. These metrics are widely used in population genetics and are particularly suitable for microsatellite data (Weir and Cockerham 1984; Excoffier et al. 2009).

Recent studies have highlighted the importance of considering both historical and contemporary factors influencing population differentiation. For example, Jombart et al. (2017) demonstrated that landscape features and anthropogenic activities can significantly alter gene flow patterns, leading to increased genetic differentiation among populations. Similarly, Paz‐Vinas et al. (2018) emphasized the role of environmental heterogeneity in shaping genetic structure, particularly in species with limited dispersal capabilities, such as sturgeons.

Inbreeding, the mating of closely related individuals, is a critical concern in small or isolated populations, as it can lead to reduced genetic diversity and increased homozygosity, potentially resulting in inbreeding depression (Charlesworth and Willis 2009). Microsatellite markers are highly effective for detecting inbreeding due to their ability to capture fine‐scale genetic variation. In our study, we estimated inbreeding coefficients (FIS) to assess the level of inbreeding within each population.

Recent advancements in genetic analysis have improved our ability to measure inbreeding more accurately. For instance, Keller et al. (2018) introduced genomic‐based approaches to estimate inbreeding coefficients, which provide higher resolution compared to traditional methods. Additionally, Huisman et al. (2016) developed software tools that integrate pedigree and genomic data to detect inbreeding and its effects on fitness‐related traits. These methods have been successfully applied to endangered species, including sturgeons, to inform conservation strategies (Bragg et al. 2020).

The findings of our study, combined with insights from recent literature, underscore the importance of maintaining genetic connectivity among sturgeon populations to prevent excessive differentiation and inbreeding. Conservation efforts should focus on preserving natural habitats, restoring migration routes and implementing genetic monitoring programmes to ensure the long‐term viability of these populations.

### 4.4 Genetic Bottleneck and STRCTURE Analysis‐Based Evidence Outputs Between Studied Populations

Furthermore, the findings from the bottleneck analysis revealed an l‐shaped pattern across all eight studied populations, confirming the results of the Arlequin analysis. This analysis assesses the probability of recent bottlenecks within the sample under examination. Populations that have undergone a recent reduction in their effective population size typically exhibit a corresponding decrease in allelic numbers and heterozygosities at polymorphic loci. Notably, allelic diversity diminishes at a rate faster than heterozygosity, especially when the observed heterozygosity exceeds the heterozygosity expected from the observed allele number in a locus at mutation drift equilibrium.

The evidence from the STRUCTURE analysis also revealed elevated levels of allelic sharing and admixture across the eight populations under study, indirectly indicating high genetic diversity within each population and low genetic distance between them. This aligns with the results reported by Zhao et al. (2005) in the Chinese sturgeon (*A. sinensis* Grey), demonstrating no significant genetic differentiation concerning the genetic distance between samples collected in different years. Similarly, Robinson et al. (2005) noted no significant genetic or genotypic differences within the Saskatchewan River system, whereas all population samples collected in this system exhibited significant differences from those in the Winnipeg, Nelson and Rainy River systems. Furthermore, the significant genetic distinctions among lake sturgeon populations in the Rainy, Saskatchewan, Winnipeg and Nelson river systems highlight the presence of distinct genetic stocks within each of these river systems. Additionally, Barmintseva and Mugue (2013) conducted a study on Russian and Persian sturgeon species, demonstrating a high probability of accurately assigning each individual to its species based on genotyping with five microsatellite loci, achieving an average accuracy level of 96%–98%. These findings are consistent with previous studies. Homola et al. (2010) examined the genetic diversity of sea sturgeon (*A*cipenser *fulvescens*), revealing a high level of genetic divergence between populations. The results of probability‐based attribution tests indicated that no lake sturgeon from the Lake Winnebago tribe had strayed from the St. Louis River into the Sturgeon River spawning population. In a study by Ludwig and Kirschbaum (1998) on the genetic diversity of Atlantic sturgeon in the Baltic Sea using SSR, it was described that the Baltic population of *Acipenser oxyrinchus* descended from a relatively small number of founders originating from the northern part of the species richness in North America. Similarly, Dudu et al. (2008) reported a low level of polymorphism in the population of sturgeons studied in the Romanian sturgeon species.

Understanding the biology and reproduction of aquatic animals, such as sturgeons, and their spawning and migration patterns is crucial for the preservation of their diversity. The genetic diversity and population genetic structure in sturgeon are influenced by factors such as geographic location, environmental conditions, selective forces, the status of primary reserves and hybridization and interaction between farmed and wild populations (Wang et al. 2022; Dudu et al. 2014). Maintaining genetic diversity is imperative, as reduced genetic diversity can render populations unable to adapt to environmental stresses such as climate change or changes in available resources, as the underlying genetic diversity for selection may have already been depleted from the population (Zhao et al. 2005; Wang et al. 2022). Heterozygotes carry different alleles, indicating diversity and adaptability under varying environmental conditions, encompassing numerous economic, fertility and disease‐resistance traits. The number of alleles and heterozygosity can also be influenced by the sample size and the target region (Zhao et al. 2005). Research on genetic diversity, genetic variation in microsatellite sites and inbreeding rates can provide valuable information for the conservation of aquatic resources in our country and offer appropriate solutions (Zhang et al. 2013). Understanding the genetic diversity of sturgeons is fundamental for genetic conservation and the species’ ability to adapt to environments impacted by environmental stresses. Knowledge of individual diversity within a species can aid in implementing sustainable fishing measures, establishing stock and brood management protocols and maintaining the stability and dynamics of aquatic populations.

In population genetics and molecular diversity studies of sturgeon species, such as beluga (*H. huso*) and other sturgeons, microsatellite markers provide critical data on genetic diversity, heterozygosity, inbreeding and genetic distance. These parameters are invaluable for designing practical and effective conservation strategies, particularly for broodstock populations maintained in hatcheries or aquaculture facilities. High genetic diversity and heterozygosity, as revealed by microsatellite analysis, are essential for ensuring a population's adaptive potential and resilience to environmental changes. In captive broodstock, maintaining genetic diversity is crucial to preserve adaptive alleles and support long‐term survival. Microsatellite data can identify populations with low genetic variability, enabling targeted interventions such as introducing individuals from wild or genetically distinct populations to enhance diversity.

Inbreeding, measured through microsatellite markers, is a significant concern in small or isolated populations. High inbreeding levels can lead to reduced fitness, lower survival rates and decreased reproductive success. To mitigate these effects, breeding programmes should use microsatellite data to select unrelated individuals for mating, minimizing inbreeding and its negative consequences. If inbreeding levels are high, microsatellite analysis can guide the introduction of new genetic material from wild populations or other hatcheries, restoring genetic health and improving population viability.

Genetic distance metrics, such as FST derived from microsatellite data, help identify distinct genetic units and inform management decisions. Populations with significant genetic differentiation should be managed separately to preserve their unique genetic heritage. Microsatellite data can also guide translocation or supplementation programmes, ensuring that genetically similar populations are used to avoid outbreeding depression. In captive breeding programmes, these metrics help select suitable broodstock pairs to maintain or enhance genetic diversity while avoiding excessive differentiation.

Practical conservation strategies based on microsatellite data include designing breeding programmes that maximize genetic diversity and minimize inbreeding, regularly updating broodstock with individuals from wild populations and implementing long‐term genetic monitoring programmes to track changes in genetic parameters over time. Additionally, restoring natural habitats and migration routes is critical for supporting wild sturgeon populations and reducing reliance on captive breeding. Microsatellite data can identify populations that would benefit the most from habitat restoration or managed translocations, promoting genetic connectivity and enhancing resilience.

By integrating microsatellite data with practical management actions, conservationists can develop targeted strategies to maintain healthy broodstock populations, enhance genetic resilience and ensure the long‐term survival of these endangered species. This approach supports not only the conservation of sturgeon populations but also contributes to the broader goals of biodiversity preservation and ecosystem health.

We acknowledge that sample size is a critical factor in population genetic studies, as it can influence the accuracy and reliability of statistical measures such as within‐population genetic diversity (e.g., observed heterozygosity, expected heterozygosity and allelic richness) and between‐population differentiation (e.g., FST and GST).

Small sample sizes can lead to underestimation of genetic diversity, particularly allelic richness, as rare alleles may not be detected (Hale et al. 2012; Allendorf et al. 2013). This is because smaller samples are less likely to capture the full spectrum of genetic variation in a population. Additionally, small sample sizes can increase the variance of genetic diversity estimates, reducing the statistical power to detect significant differences between populations.

In our study, we carefully considered these limitations during the design phase. Although our sample size may appear modest, it is consistent with the challenges of working with endangered species such as sturgeons, where obtaining large sample sizes is often logistically and ethically constrained (Birstein et al. 1997; Ludwig et al. 2009). To mitigate the potential biases associated with small sample sizes, we employed robust statistical approaches, such as rarefaction analysis, to standardize allelic richness across populations (Kalinowski 2004). Furthermore, we used bootstrapping methods to assess the stability of our FST estimates and confirmed that our results are consistent and reliable despite the sample size limitations.

Recent studies have also highlighted the importance of considering the effects of sample size in genetic diversity assessments. For example, Hoban et al. (2020) emphasized that although larger sample sizes are ideal, meaningful genetic insights can still be obtained from smaller samples if appropriate analytical methods are applied. Similarly, Nazareno et al. (2017) demonstrated that small sample sizes can still provide accurate estimates of genetic differentiation when populations are highly structured.

In conclusion, although we recognize the limitations imposed by our sample size, we are confident that our findings provide valuable insights into the genetic diversity and structure of the studied sturgeon populations. Future studies with larger sample sizes would further enhance our understanding of these populations.

This study is subject to certain limitations. The analysis of the pattern and variability of microsatellite markers was restricted to only eight populations, and a relatively small sample size was used for each population. Horizontal electrophoresis involving a metaphor agarose gel may also have introduced certain limitations to the analysis. It is important to note that genotyping errors, particularly related to stutter bands, are inherent to microsatellite marker genotyping. Moreover, the use of a limited set of SSRs may have implications for the robustness of genetic diversity assessments. Given these limitations, we recommend high‐tech next‐generation sequencing (NGS) and SNP chip studies to investigate the inbreeding rate within broodstock‐based populations.

To address the limitations identified in this study and enhance the quality and reliability of future research, we propose a series of targeted recommendations. First, expanding the scope of the study to include a greater number of populations and increasing the sample size within each population would significantly improve the accuracy and representativeness of genetic diversity estimates. Larger sample sizes reduce sampling bias and increase the statistical power of analyses, enabling more robust conclusions about population structure and genetic variation. Additionally, adopting advanced genotyping techniques, such as CE, is essential to overcome the resolution limitations associated with traditional methods like metaphor agarose gel electrophoresis. Automated genotyping systems should also be employed to minimize human error and enhance the precision of data collection.

To further strengthen genetic analyses, it is recommended to increase the number of microsatellite markers used and to integrate them with other types of genetic markers, such as SNPs. This multi‐marker approach would provide a more comprehensive understanding of genetic diversity and population differentiation. Specialized software tools should be utilized to correct genotyping errors, particularly those related to stutter bands, and experimental replicates should be conducted to ensure the reproducibility and reliability of results.

For a more advanced and detailed analysis, NGS and SNP chip technologies are highly recommended. NGS allows for the simultaneous examination of thousands of genetic markers, offering unparalleled resolution for estimating genetic diversity, population structure and inbreeding rates. SNP chips, on the other hand, provide a cost‐effective and efficient means of analysing large datasets, making them ideal for studies involving multiple populations. These technologies not only improve the accuracy of genetic assessments but also enable the identification of fine‐scale genetic patterns that may be missed by traditional methods.

Finally, to ensure the highest standards of research, it is crucial to adhere to international guidelines for genetic studies, foster collaborations with international research institutions to access larger datasets and advanced technologies and implement robust data management systems. These steps will enhance comparability, reproducibility and overall quality of future studies. By adopting these strategies, researchers can overcome the limitations of current methodologies, generate more reliable and insightful results and contribute to the development of effective conservation and management strategies for aquatic populations.

## 5 Conclusion

Genetic characterization is a vital initial step in designing effective management and conservation programmes for aquatic animals in developing countries. The analysis of genetic diversity among the eight populations studied revealed notable levels of allelic sharing and allelic admixture, indirectly indicating high genetic diversity within individual populations and low genetic distance between them. Specifically, our results identified the families LS19 (*n_a_* = 16) and Afu54 (*n_a_* = 3) as displaying the highest and lowest polymorphisms, respectively. Additionally, our assessment demonstrated that the IFRO farm population exhibited the highest genetic diversity (Ave_Het = 0.67), whereas the Rajaeei Sturgeon Private Farm showed the lowest diversity score (Ave_Het = 0.55) among the populations studied. Furthermore, our findings indicated that the INS and Jahanpouri Sturgeon Private Farm exhibited the highest similarity (similarity 0.91), whereas the Saeei Sturgeon Private Farm displayed the lowest genetic distance among the populations studied (similarity 0.74). The STRUCTURE analysis reinforced these results, highlighting considerable levels of allelic sharing and admixture across the studied populations, indirectly indicating the presence of genetic diversity within each population and low genetic distance between them. These findings underscore the importance of understanding DNA variability and population structure as essential components of any conservation programme.

## Author Contributions

**Mehdi Moghim**: conceptualization, methodology, investigation, data curation, formal analysis, writing – original draft, writing – review and editing, supervision, project administration, funding acquisition. **Arash Javanmard**: conceptualization, methodology, investigation, data curation, writing – review and editing. **Faramarz Lolaei**: conceptualization, methodology, investigation, data curation, writing – review and editing. **Mohammad‐Javad Taghavi**: conceptualization, methodology, investigation, data curation, writing – review and editing. **Shima Bakhshalizadeh**: conceptualization, methodology, investigation, data curation, writing – review and editing.

## Ethics Statement

Animal care and experimental research were in accordance with the “Position on the Ethics of Using Animals in Research supported by the Russian Science Foundation” and The Guide for the Care and Use of Laboratory Animals (National Academy Press, Washington, D.C., 1996). Efforts were made to minimize the suffering of animals and reduce the number of samples used (Protocol 1 of 05/21/2021).

## Conflicts of Interest

The authors declare no conflicts of interest.

### Peer Review

The peer review history for this article is available at https://publons.com/publon/10.1002/vms3.70255

## Data Availability

Data are available from the corresponding author upon reasonable request.
